# Microstructure Evolution and Mechanical Properties of Al–Cu–Mg Alloys with Si Addition

**DOI:** 10.3390/ma16072783

**Published:** 2023-03-30

**Authors:** Abdul Wahid Shah, Seong-Ho Ha, Jabir Ali Siddique, Bong-Hwan Kim, Young-Ok Yoon, Hyun-Kyu Lim, Shae K. Kim

**Affiliations:** 1 Industrial Technology, University of Science and Technology, Daejeon 34113, Republic of Korea; abdulwahid.shah799@gmail.com (A.W.S.);; 2 Industrial Materials Processing R&D Department, Korea Institute of Industrial Technology, Incheon 21999, Republic of Korea

**Keywords:** Al–Cu–Mg alloy, Si addition, microstructure, heat treatment, tensile properties

## Abstract

The aim of this study was to investigate the impact of the addition of a minor quantity of Si on the microstructure evolution, heat treatment response, and mechanical properties of the Al–4.5Cu–0.15Ti–3.0Mg alloy. The microstructure analysis of the base alloy revealed the presence of α-Al grains, eutectic α-Al-Al_2_CuMg (S) phases, and Mg_32_(Al, Cu)_49_ (T) phases within the Al grains. In contrast, the Si-added alloy featured the eutectic α-Al-Mg_2_Si phases, eutectic α-Al-S-Mg_2_Si, and Ti-Si-based intermetallic compounds in addition to the aforementioned phases. The study found that the Si-added alloy had a greater quantity of T phase in comparison to the base alloy, which was attributed to the promotion of T phase precipitation facilitated by the inclusion of Si. Additionally, Si facilitated the formation of S phase during aging treatment, thereby accelerating the precipitation-hardening response of the Si-added alloy. The as-cast temper of the base alloy displayed a yield strength of roughly 153 MPa, which increased to 170 MPa in the Si-added alloy. As a result of the aging treatment, both alloys exhibited a notable increase in tensile strength, which was ascribed to the precipitation of S phases. In the T6 temper, the base alloy exhibited a yield strength of 270 MPa, while the Si-added alloy exhibited a significantly higher yield strength of 324 MPa. This novel Si-added alloy demonstrated superior tensile properties compared to many commercially available high-Mg-added Al–Cu–Mg alloys, making it a potential replacement for such alloys in various applications within the aerospace and automotive industries.

## 1. Introduction

Aluminum–copper–magnesium (Al–Cu–Mg)-based alloys, such as A201 and A206, are the strongest casting alloys of aluminum [1]. The copper content in these alloys varies between 4 and 10% by weight, with most alloys containing around 4.5%. Magnesium is an essential component of these alloys, and depending on the amount of magnesium present, they can be categorized into low-magnesium- and high-magnesium-containing Al–Cu–Mg alloys [1,2]. Aluminum alloys such as A201 and A206, which have a magnesium content below 1%, are renowned for their high strength and toughness. They also possess excellent corrosion resistance, and their precipitation hardening during heat treatment results in the highest tensile strength among all aluminum casting alloys [1,2,3,4,5]. However, these alloys have some disadvantages, including a relatively high tendency to hot tearing and low corrosion resistance [6,7,8,9,10]. Alloys with higher magnesium content (1.5~6%), such as 240, 242, and 243 alloys, are renowned for their superior hardness, high specific strength, and thermal stability at elevated temperatures. These alloys are commonly employed in applications where wear resistance and thermal stability are crucial factors, such as the production of pistons for internal combustion engines (e.g., 242 and A242 alloys) and air-cooled cylinder heads for aircraft engines [1]. Alloys with high magnesium content (such as 240, 242, and 243) exhibit better fluidity and resistance to hot tearing than those with low magnesium content (such as A201 and A206) [1,2].

In the Al–Cu–Mg ternary phase diagram, the Al_2_CuMg (S) intermetallic compound (IMC) is comparable to Mg_2_Si in the Al–Mg–Si ternary phase diagram. It divides the diagram into two sections on the Al-rich corner: α-Al + Al_2_Cu (θ) + S and α-Al + Mg_32_(Al, Cu)_49_ (T) + S. A quasi-binary eutectic reaction (L => α-Al + S) occurs at a Cu/Mg ratio of 2.40, resulting in various non-variant reactions in the Al-rich corner of Al–Cu–Mg ternary alloys [2]. Low-Mg-containing alloys in the α-Al + θ + S section of the diagram are primarily composed of a primary α-Al matrix and eutectic α-Al-θ phases, along with a small amount of eutectic α-Al-S phases [3,5,11,12,13,14,15,16,17,18,19,20]. High-Mg-containing Al–Cu–Mg commercial alloys have compositions lying in the α-Al + S + θ and α-Al + S sections of the diagram [1,2,19,20,21]. However, due to their higher Mg content, these alloys have a significantly greater amount of eutectic α-Al-S phases than low-Mg-containing alloys.

However, the development of Al–Cu–Mg alloys with compositions in the α-Al + S + T section of the Al–Cu–Mg ternary phase diagram has received very little attention so far, according to the authors’ knowledge [19,20,21]. High-Mg-containing Al–Cu–Mg alloys exhibit significantly higher tensile strength compared to Al–Si-based commercial alloys in the as-cast condition. However, compared to other heat-treated Al alloys such as A356 and A206 alloys, their response to heat treatment is less effective [1]. This is because the high Mg content leads to the formation of a higher amount of S phases in the as-cast microstructure, which do not dissolve during solution heat treatment, thus decreasing the heat treatment response of these alloys [20]. However, selecting a composition within the α-Al + S + T section of the Al–Mg–Cu ternary phase diagram results in the formation of T precipitates that can be dissolved during solution heat treatment, thus improving the precipitation hardening of high-Mg-containing alloys. In addition, the incorporation of minor quantities of other elements (such as Si, Ag, Mn, Zn, Ge, and Sn) has been reported to have a significant impact on the microstructure evolution and heat treatment response of Al–Cu–Mg alloys with low Mg content [11,12,13,14,15,16,17,18,19,21]. Despite extensive studies on Al–Cu–Mg alloys with low Mg content, there has been limited research on how alterations in chemical composition impact the microstructure evolution and heat treatment response of Al–Cu–Mg alloys that contain a high Mg content.

The enhancement in tensile strength through the development of new Al–Cu–Mg alloys with high Mg content is of great importance to the aerospace and automotive industries, given their good thermal stability at elevated temperatures. The primary objective of this study was to modify the chemical composition of these alloys to enhance their strength. Specifically, the study aimed to investigate the impact of adding small amounts of other elements to the Al–Cu–Mg alloys with high magnesium content, located in the α-Al + S + T section of the Al–Mg–Cu ternary phase diagram. The study focused on analyzing the solidification behavior, microstructure evolution, heat treatment response, and mechanical properties of the Al–4.5Cu–0.15Ti–3.0Mg alloy with the inclusion of a small amount of Si. 

## 2. Materials and Methods

The chemical composition of the experimental alloys utilized in the investigation is presented in Table 1. The base material employed in the study was high-purity aluminum ingots with a purity level of 99.99%. To form the experimental alloys, copper, magnesium, titanium, and silicon were introduced into the melt through the use of Al–50%Cu, Mg + Al_2_Ca, Al–5%Ti–1%B, and Al–25%Si master alloys, respectively. The melting process involved melting an aluminum ingot in an induction furnace at ambient atmosphere, followed by alloying at temperatures of 760–800 °C. After melting the pure aluminum, the melt was maintained at around 750 °C for a brief duration to ensure the uniform distribution of the alloying elements. To remove hydrogen gas and oxide inclusions, gas bubbling filtration (with Ar gas) was utilized for a duration of 15 min. The temperature was kept at approximately 700 °C throughout the degassing process. After degassing, the melt was held at approximately 690 °C for 5 min before being poured into a preheated steel mold at the same temperature for all alloys. The experimental alloys underwent a two-step heat treatment. The first step was a solution heat treatment at a temperature of 477 °C for 10 h, followed by water quenching. The second step was an aging treatment, which was carried out at 200 °C for 20 h. For the hardness measurements at various aging times, the Brinell hardness machine from Buehler (Uzwil, Switzerland) was employed. For each condition, five measurements were taken for every specimen, and the mean of these five measurements is presented.

The theoretical calculations were performed using JMatPro 11.2 software. The differential scanning calorimetry (DSC, TA Q1000 instrument, TA instruments, Milford, MA, USA) experiments involved heating the samples of each alloy in an alumina pan within a furnace, and the specimens were heated at a rate of 10 °C per minute in an argon atmosphere ranging from 100 °C to 700 °C. For the precipitation behavior of the investigated alloys, the as-quenched samples were heated between 30 °C and 580 °C under an argon atmosphere at a rate of 10 °C per minute while placed in pure aluminum pans in the furnace. The microstructure of the experimental alloys was examined using optical microscopy (OM, Nikon, Tokyo, Japan) and field emission-scanning electron microscopy (FE-SEM, FEI model Quanta 200 F) with energy-dispersive spectroscopy (EDS, EDAX, Pleasanton, CA, USA). Prior to OM observation, the samples were prepared by grinding, micro-polishing, and etching in Keller’s reagent. FE-SEM analysis was conducted under specific conditions, including an accelerating voltage of 20 KV and a working distance of 10.0 mm. The tensile samples were prepared as per ASTM standard B557 and tested using a universal tensile testing machine (DTU-900MHN, Daekyung Tech, Gumisi, Republic of Korea) with a strain rate of 1.5 mm/min and an extensometer gauge length of 30 mm.

## 3. Results and Discussion

**Figure 1:**
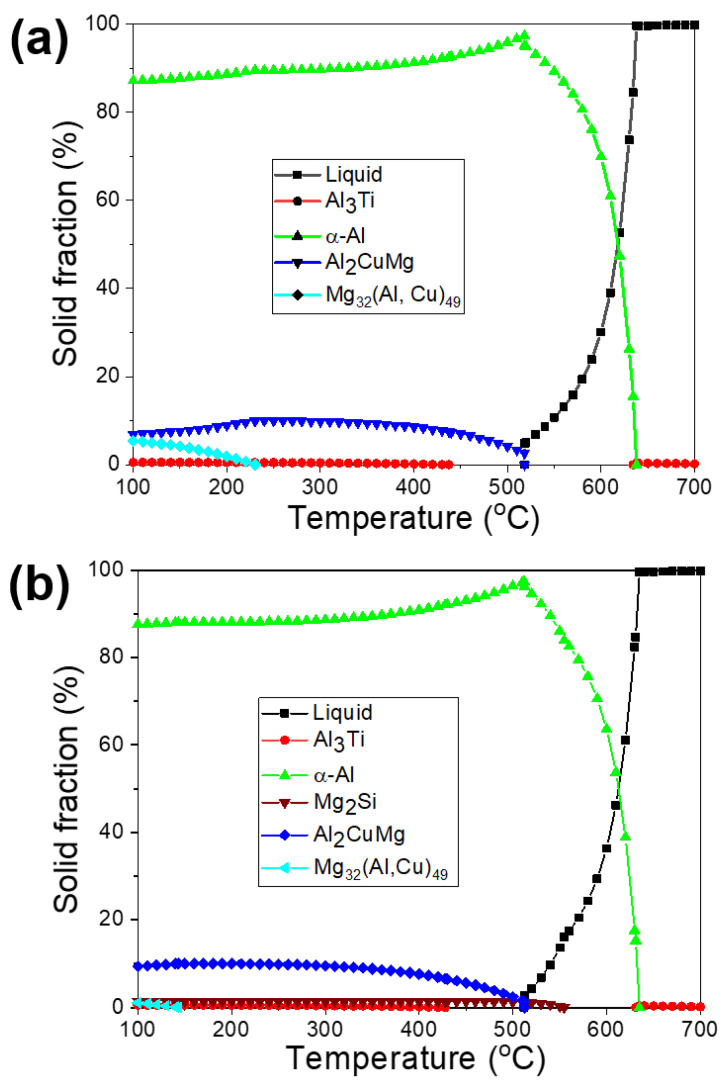
Results of theoretical calculations conducted using JMatPro 7.0 to evaluate different phases during the solidification process of the B43 and S43 alloys, as shown in (**a**) and (**b**), respectively.

Table 2 shows all of the non-variable eutectic reactions that have taken place on the aluminum-rich corner of the Al–Cu–Mg–Si quaternary phase diagram [2]. The introduction of a small quantity of silicon into the Al–Cu–Mg ternary system results in the development of Mg_2_Si phases through either a ternary reaction (L => α-Al + S + Mg_2_Si) or a quaternary reaction (L => α-Al + Al_2_Cu + Al_2_CuMg (S) + Mg_2_Si), depending on the ratio of Cu to Mg and the amount of Si (as presented in Table 2). In this study, theoretical calculations were conducted using JMatPro 7.0 software (based on equilibrium cooling) to explore the formation of different phases in the alloys examined during the solidification process. Based on the theoretical calculations shown in Figure 1a, it is predicted that the B43 (Al–4.5Cu–0.15Ti–3.0Mg) alloy consists of α-Al, eutectic α-Al-S, Al_3_Ti intermetallic compounds (IMCs), and T phases. A univariant eutectic (L => α-Al + S) reaction is predicted to end the solidification process in this alloy. Additionally, it is predicted that the T phases precipitate out once the solidification process is completed and the temperature decreases to 250 °C. On the other hand, apart from the phases observed in the B43, the formation of Mg_2_Si phases is also predicted in the S43 (Al–4.5Cu–0.15Ti–3.0Mg–0.5Si) alloy (Figure 1b) through the following univariant reaction: L => α-Al + Mg_2_Si at around 560 °C. Furthermore, it is predicted that the addition of silicon decreases the precipitation temperature of the T phases to below 150 °C and reduces their amount compared to the B43 base alloy (Figure 1a). A decrease in the amount of T phases in the Si-added alloy (S43) is linked to the consumption of magnesium in the creation of eutectic α-Al-Mg_2_Si phases, resulting in a lower amount of magnesium within the Al matrix for the precipitation of T phases.

**Figure 2:**
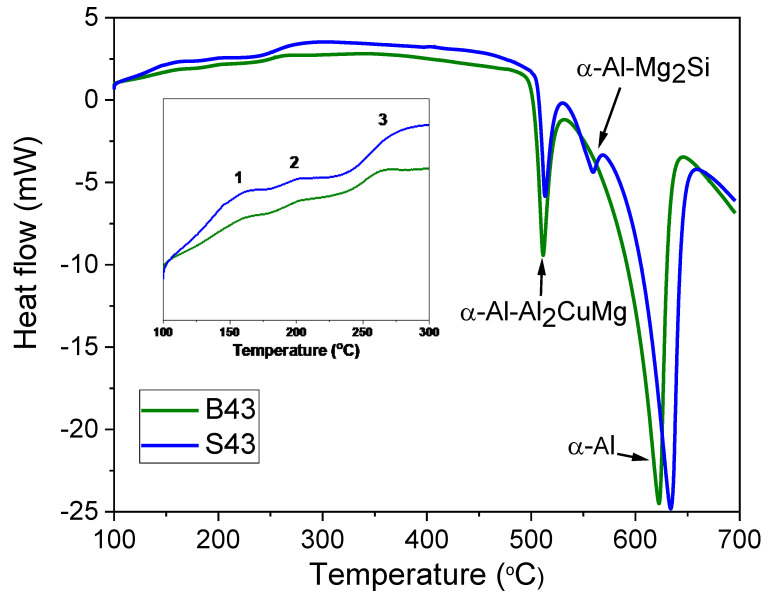
Differential scanning calorimetry (DSC) curves of the investigated alloys, which were heated at a rate of 10 °C per minute.

Figure 2 shows the DSC results of the B43 and S43 alloys, revealing solidus temperatures of approximately 493 °C and 500 °C, respectively. In the B43 alloy, a eutectic peak was detected in addition to the peak related to the α-Al matrix, indicating the formation of a quasi-binary eutectic reaction (L => α-Al + S). The S43 alloy exhibited an additional peak, likely indicating the formation of binary eutectic (α-Al-Mg_2_Si) phases due to the addition of 0.5%Si. These results suggest that the solidification process in the B43 base alloy ends with an univariant binary (α-Al-S) reaction, while in the S43 alloy, solidification ends with either a binary (α-Al-S) or a ternary (α-Al-Mg_2_Si-S) eutectic reaction (as per Table 2). Both alloys showed exothermic peaks below 300 °C, with peak #1 likely indicating the precipitation of T phases, consistent with the phase diagram in Figure 1. Notably, the formation temperatures of both alloys were almost identical, contrary to the theoretical calculations. Peak #2 may be linked to the formation of GPB zones or solute clusters. Peak #3 observed between 250 and 280 °C could be associated with the precipitation of S″ or S′ phases, as suggested in prior research [20,21,22,23].

**Figure 3:**
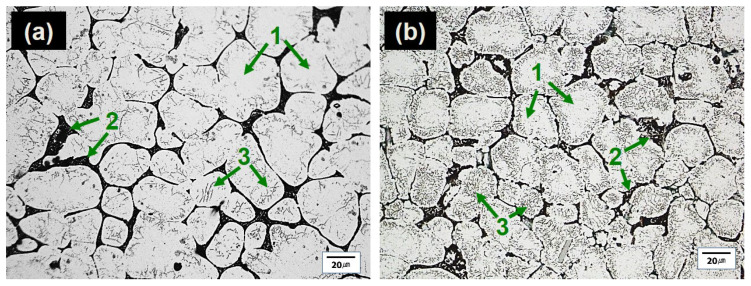
Optical micrographs of the investigated alloys in their as-cast condition. The micrographs show (**a**) the B43 alloy and (**b**) the S43 alloy. Here, (1), (2), and (3) indicate the α-Al grains, eutectic Al-S/Al-S and Al-Mg_2_Si phases, and T precipitates, respectively.

**Figure 4:**
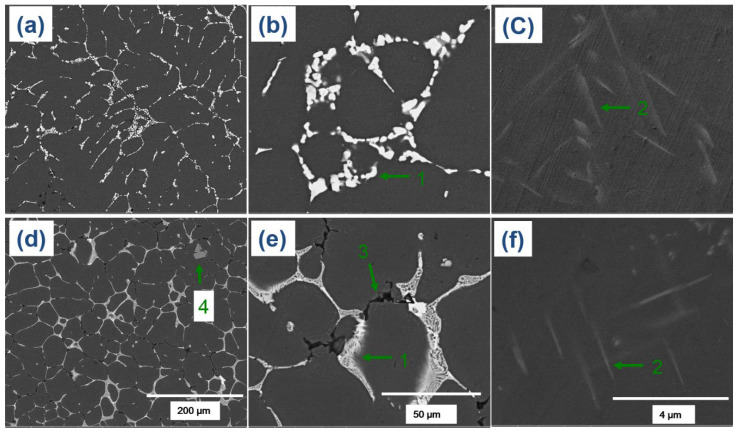
FE-SEM micrographs of the investigated alloys in their as-cast condition. The microstructures of the B43 alloy are shown in (**a**–**c**), while (**d**–**f)** show the microstructures of the S43 alloy. The annotations (1), (2), (3), and (4) indicate the eutectic Al-S, T precipitates, eutectic Al-Mg_2_Si, and Ti-based intermetallic phases, respectively.

**Figure 5:**
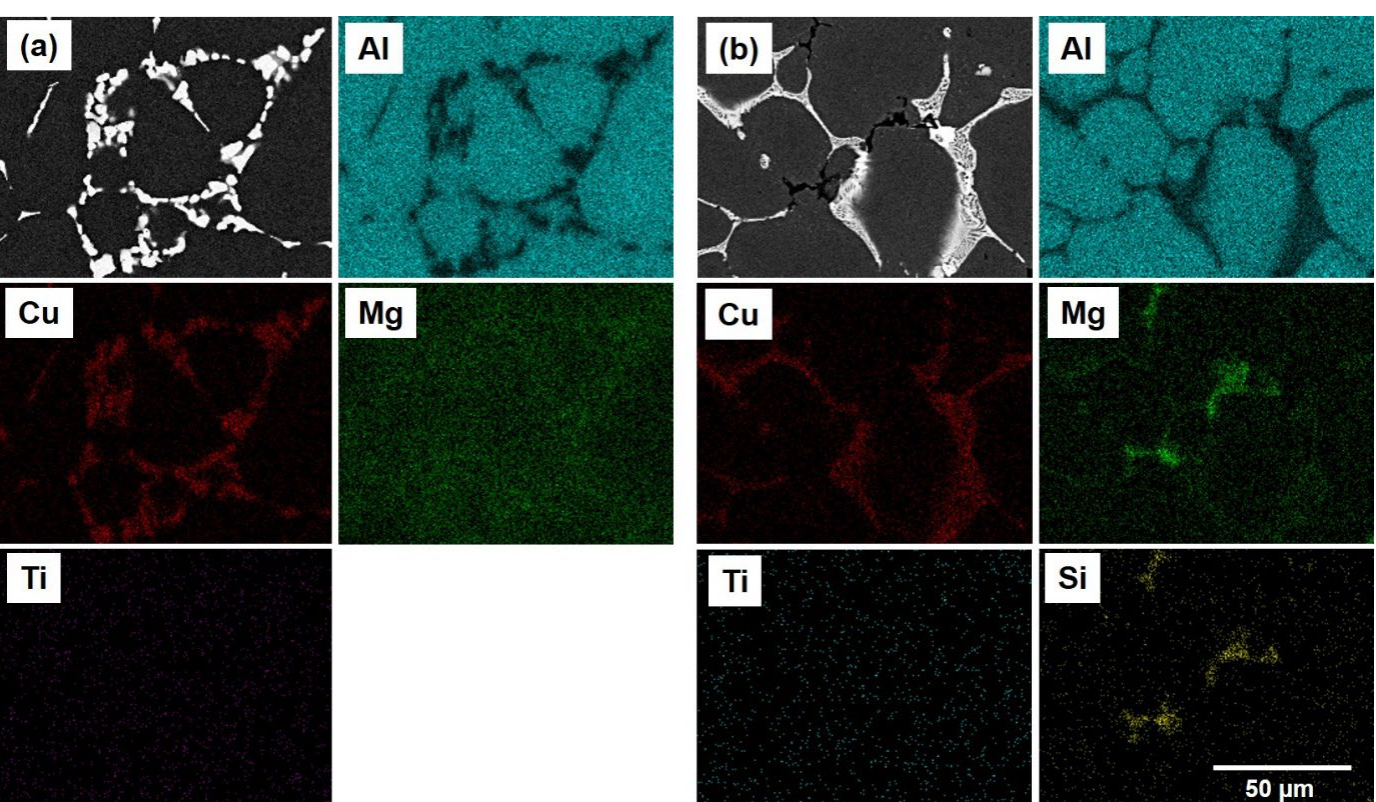
FE-SEM micrographs of (**a**) the B43 alloy and (**b**) the S43 alloy, along with their corresponding EDS mapping analyses.

The optical microscopy (OM) micrographs of the investigated alloys showed that the microstructures primarily consisted of globular grains of α-Al matrix with eutectic α-Al-S or α-Al-S and α-Al-S-Mg_2_Si phases at the grain boundaries and T precipitates within the α-Al matrix, as seen in Figure 3. The globular grains present in the B43 alloy had a size of 36.4 µm, which was reduced to 29.7 µm in the S43 alloy. The size of the globular grains in the S43 alloy (Figure 3b) was slightly smaller than that in the base alloy (Figure 3a) due to the addition of silicon, which increased the solute concentration on the solid/liquid interface and the formation of binary eutectic α-Al-Mg_2_Si phases at higher temperature, suppressing the grain growth. The scanning electron microscopy (SEM) analysis shown in Figure 4 and Figure 5 confirmed that the microstructure of the base alloy consisted of α-Al grains (#1), eutectic α-Al-S phases (#2), and T phases (#3) within the Al grains. In contrast, the Si-added alloy featured the eutectic α-Al-Mg_2_Si phases, eutectic α-Al-S-Mg_2_Si, and Ti-Si based intermetallic compounds (#4) in addition to the aforementioned phases. The SEM-EDS analysis of the second phases also confirmed that the eutectic Al-Mg_2_Si phases were formed through an univariant eutectic (L => α-Al + Mg_2_Si) reaction. The elemental composition of these second phases was determined by SEM-EDS analysis. The morphology of eutectic phases (Figure 4a,b) was significantly changed with the addition of silicon (Figure 4d,e), indicating that an univariant eutectic (α-Al-S-Mg_2_Si) reaction ended solidification in the S43 alloy instead of the quasi-binary eutectic (L => α-Al + S) reaction. The addition of Si also caused the formation of coarse Ti-Si based IMCs, which are undesirable as they decrease the grain refinement efficiency of Ti by decreasing the amount of free Ti in the alloy melt.

**Figure 6:**
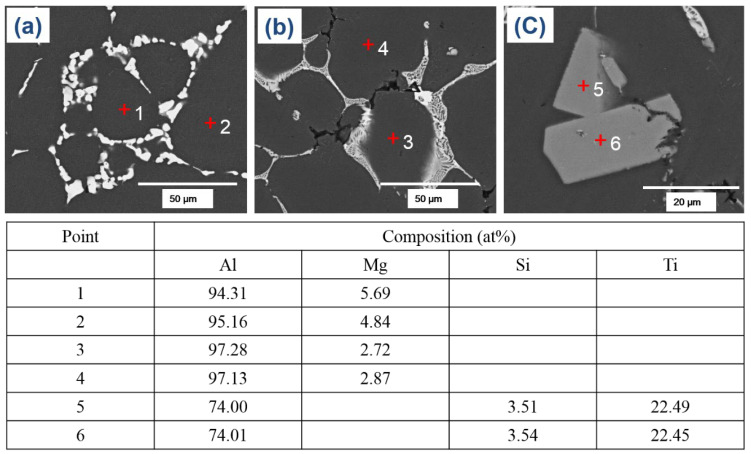
FE-SEM micrographs of (**a**) the B43 alloy and (**b**,**c**) the S43 alloy in their as-cast condition. The table below the micrographs provides the composition of the points indicated in the micrographs.

The T phases (#2) were formed (Figure 4c,f) during the solidification process, when the temperature fell below the solidus temperature, as mentioned earlier. Although the theoretical calculations in Figure 1 showed that the addition of Si decreased the amount of T precipitates, the OM micrographs of the B43 alloy (Figure 3a) and S43 alloy (Figure 3b) revealed a noteworthy rise in the amount of T phase in the Si-added alloy. The Al matrix in the B43 alloy contained approximately 5.0 at% Mg (#1 and #2), which decreased to about 2.80 at% (#3 and #4) in the S43 alloy, as shown in Figure 6. This reduction in the amount of Mg contents in the Al matrix can be related to the formation of eutectic α-Al-Mg_2_Si phases and an increasing amount of T precipitates. Previous studies on Si addition in Al–Cu–Mg with a low Mg/Cu ratio showed that Si addition significantly reduced the dislocation density and stabilized GPB zones, resulting in a more uniform distribution of fine precipitates than Si-free alloys [19,21]. A similar mechanism is more likely in the current Si-added alloy, leading to a significant increase in the amount of T phase in Figure 3b. Therefore, it can be concluded that the addition of Si encourages the precipitation of T phases within the Al matrix.

**Figure 7:**
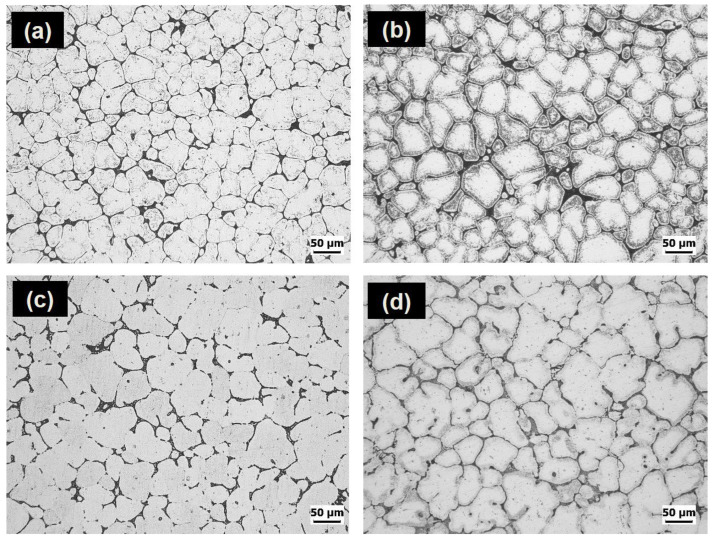
Optical micrographs of the investigated alloys in different temper conditions. Microstructures of the B43 alloy are displayed in (**a**,**c**), while (**b**,**d**) show the as-cast and as-quenched microstructures of the S43 alloy, respectively.

**Figure 8:**
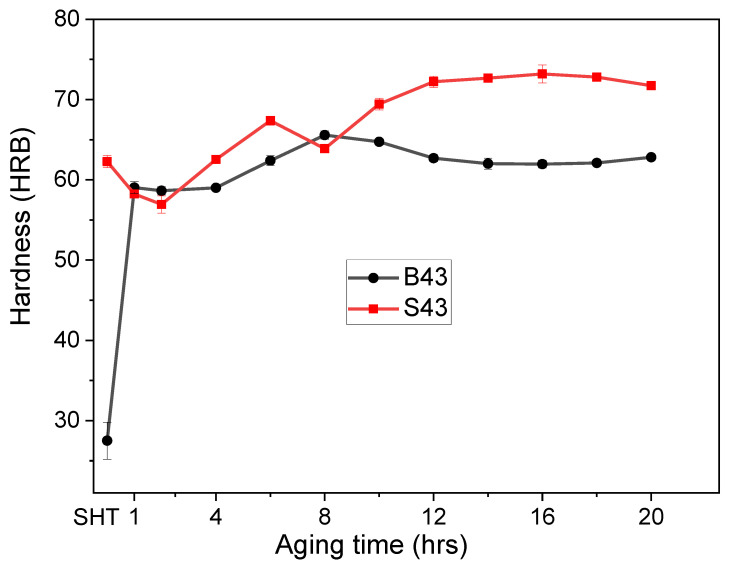
Change in the hardness values for both examined alloys as a function of aging time.

Figure 7 displays the OM micrographs of the investigated alloys in both the as-cast and as-quenched tempers. The investigation revealed that very little modification in the eutectic α-Al-S, α-Al-Mg_2_Si, and α-Al-S-Mg_2_Si phases occurred upon exposing these alloys to the solution heat treatment, causing a significant amount of remnant phases in the as-quenched alloys (Figure 7c,d). However, a considerable dissolution of T precipitates was achieved after the solution heat treatment. Figure 8 shows the variation in hardness values against aging time for the investigated alloys. There was a considerable difference in the hardness of the alloys in the as-quenched states, with B43 and S43 alloys exhibiting hardness values of 27 and 62 HBR, respectively. This difference can be ascribed to the existence of Mg_2_Si phases in the S43 alloy, which did not dissolve during the solution treatment, thus maintaining the same yield strength achieved in the as-cast condition. Nonetheless, a significant increase in the hardness of the B43 alloy was observed after only 1 h of aging treatment, reaching a peak hardness of 65 HRB after 8 h of aging treatment. Thereafter, a slight decrease in hardness was observed before becoming almost constant for the rest of the aging time. In contrast, the S43 alloy initially exhibited a decrease in hardness values for up to 2 h before bouncing back upward. Upon further exposure of the S43 alloy at 200 °C, the hardness slightly decreased before starting to increase again and reaching a peak hardness value of ~73 HRB at ~16 h of aging treatment. These results clearly reveal that the minor Si addition in the S43 alloy resulted in a more noteworthy increase in the peak hardness value than the B43 base alloy.

The existing literature [19,20,21,22,23] suggests that as-quenched Al–Cu–Mg alloys consist of a supersaturated solid solution (SSSS), which is a high-energy state that transforms into GPB zones as the first stage of precipitation hardening. The growth in these GPB zones leads to the formation of the S″ metastable phase, which subsequently converts to another metastable phase (S′ phase) over time. Eventually, the S phase, which represents the stable phase, is formed through the growth of the S′ phase. While this sequence is most commonly reported, some studies [21,22,23] have reported solute clusters as the starting point for precipitate formation instead of GPB zones. In these cases, atomic clusters are observed as the first stage of precipitate formation, which are distinct from GPB zones as they lack well-defined characteristics such as proper shape, composition, and crystal structure. Regarding the evolution of hardness during the aging treatment, prior research [20,21,22,23] has identified two distinct peaks in Al–Mg–Cu alloys. The initial peak in hardness is linked to the formation of atomic clusters or GPB zones, which account for up to 60% of the total hardening attained during aging. The subsequent precipitation of S″ and S phases corresponds to the second stage of precipitation hardening, occurring later in the aging process.

**Figure 9:**
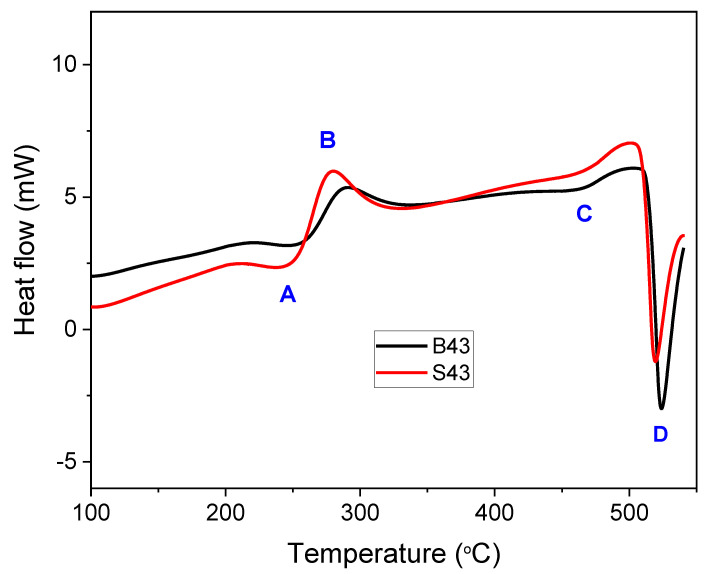
DSC curves of the investigated alloys, which were heated at a rate of 10 °C per minute.

The DSC analysis (Figure 9) of the as-quenched alloys showed three distinct endothermic peaks (**A**, **C**, and **D**) and one exothermic peak (**B**) for both B43 and S43 alloys. Previous studies on Al–Cu–Mg alloys with high Mg content have also reported similar DSC curves [20,21,22]. According to these studies, endothermic peak **A** is related to the dissolution of atomic clusters (GPB/S″/T phases), while peaks **C** and **D** represent the dissolution of S precipitates and partial melting of S + T phases, respectively. Exothermic peak **B** represents the formation of S phases. The area of peak **B**, representing the formation of S phases, increased significantly in the S43 alloy compared to the B43 alloy, indicating a considerable increase in the precipitation of S phases. Previous studies have shown that the addition of Si in Al–Mg–Cu alloys enhances the precipitation of the S phase by stabilizing GPB zones, resulting in a more uniform precipitation of fine S precipitates and higher peak hardness [19,21]. Based on the current results and previous studies, it can be concluded that the higher hardness of the S43 alloy compared to the B43 alloy can be attributed to Si-induced uniform precipitation of S″/S phases in the former alloy. The Si addition in the S43 alloy is believed to have reduced the number of dislocations and forced the S phases to nucleate on homogeneously distributed GPB zones, resulting in a more uniform distribution of fine S precipitates and higher peak hardness [19,21].

**Figure 10:**
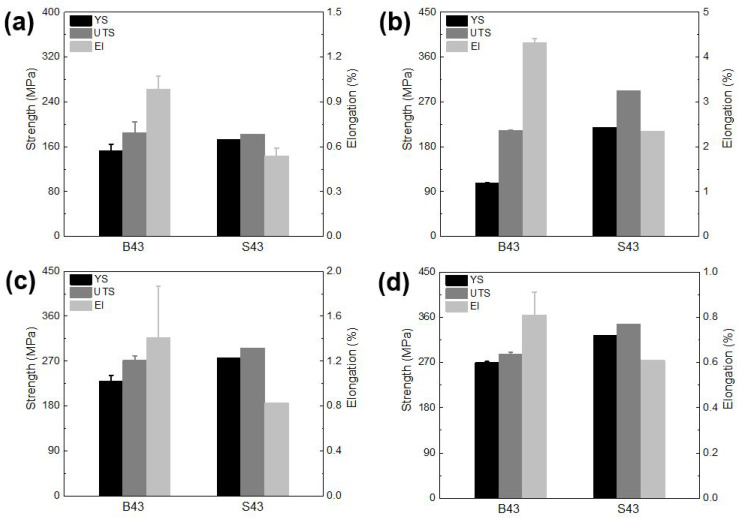
Tensile properties of the investigated alloys in different temper conditions: (**a**) as-cast, (**b**) as-quenched, (**c**) aged at 200 °C for 8 h, and (**d**) aged at 200 °C for 16 h.

Figure 10 displays the tensile properties of the investigated alloys in different tempers. In the as-cast condition, the B43 alloy achieved a yield strength of around 153 MPa, while the S43 alloy achieved a yield strength of 170 MPa (Figure 10a). This increase in tensile strength can be attributed to the presence of Mg_2_Si phases and an increasing amount of T precipitates within the Al matrix of the S43 alloy. However, this improvement in tensile strength in the S43 alloy comes at the cost of ductility, as elongation decreased to 0.5%. After undergoing the solution heat treatment process, the B43 alloy exhibited a decrease in yield strength but a significant increase in ultimate tensile strength and elongation (Figure 10b). Additionally, the S43 alloy showed a higher yield strength in the as-quenched condition compared to that achieved in the as-cast condition. This trend is similar to that reported in Al–Mg–Si ternary alloys with high Mg content, and it is attributed to the breaking of eutectic phases and redistribution of Mg_2_Si phases [24]. Furthermore, subjecting these alloys to the aging treatment at 200 °C led to a significant increase in their tensile strengths compared to those achieved in the as-cast and as-quenched conditions. These enhanced strengths were achieved with improved elongations compared to the as-cast condition. After an exposure time of 8 h, the B43 alloy reached a yield strength of 234 MPa, while the S43 alloy attained a yield strength of 245 MPa (Figure 10c). Moreover, a longer exposure time of 16 h led to a further significant enhancement in the yield strengths in both alloys, resulting in a yield strength of 270 MPa in the B43 alloy and 324 MPa in the S43 alloy (Figure 10d). However, this enhancement was associated with a decrease in elongation, which decreased to below 1% for both alloys. Nevertheless, both alloys, and the Si-added alloy in particular, exhibited much better tensile properties than many commercial alloys such as A240, A242, and A243 alloys [1].

## 4. Conclusions

This study aimed to explore how the addition of a small amount of Si impacts the microstructure evolution, heat treatment response, and mechanical properties of the Al–4.5Cu–0.15Ti–3.0Mg alloy. The following conclusions can be inferred from this research:

The microstructure analysis of the base alloy revealed the presence α-Al grains, eutectic α-Al-S phases, and T phases within the Al grains. In contrast, the Si-added alloy featured the eutectic α-Al-Mg_2_Si phases, eutectic α-Al-S-Mg_2_Si, and Ti-Si-based intermetallic compounds in addition to the aforementioned phases. The study found that the Si-added alloy had a greater quantity of T phase in comparison to the base alloy, which was attributed to the promotion of T phase precipitation facilitated by the inclusion of Si. Additionally, Si facilitated the formation of S phase during the aging treatment, thereby accelerating the precipitation hardening response of the Si-added alloy. 

The as-cast temper of the base alloy displayed a yield strength of roughly 153 MPa, which increased to 170 MPa in the Si-added alloy. As a result of the aging treatment, both alloys exhibited a notable increase in tensile strength, which was ascribed to the precipitation of S phases. In the T6 temper, the base alloy exhibited a yield strength of 270 MPa, while the Si-added alloy exhibited a significantly higher yield strength of 324 MPa. This novel Si-added alloy demonstrated superior tensile properties compared to many commercially available high Mg-added Al–Cu–Mg alloys, making it a potential replacement for such alloys in various applications within the aerospace and automotive industries.

## Figures and Tables

**Table 1:** Nominal composition of the alloys that were investigated.

Alloy	Compositions (Mass%)				
	Cu	Mg	Ti	Si	Al
B43	4.5	3.0	0.15	-	bal.
S43	4.5	3.0	0.15	0.5	bal.

**Table 2:** Non-variant reactions that are formed at the Al-rich corner of the Al–Cu–Mg-Si quaternary phase diagram, as documented in reference [2].

Eutectic Reaction	Composition (Mass%)			Temperature (°C)
	Cu	Mg	Si	
L => Al + Al_2_CuMg (quasi-binary eutectic)	24.5	10.1	-	518
L + Al_2_CuMg (S) => Al + Mg_32_(Al, Cu)_49_ (T)	10	26	-	467
L => Al + Al_2_Cu + Al_2_CuMg (S) + Mg_2_Si	33	6–7	0.3	500
L => Al + Mg_2_Si + Al_2_CuMg (S)	23	10.3	0.3	516
L + Al_2_CuMg (S) => Al + Mg_32_(Al, Cu)_49_ (T) + Mg_2_Si	10	25	0.3	467

## Data Availability

Available upon request from the corresponding author.
